# High Resolution Multimodal Photoacoustic Microscopy and Optical Coherence Tomography Visualization of Choroidal Vascular Occlusion

**DOI:** 10.3390/ijms21186508

**Published:** 2020-09-05

**Authors:** Van Phuc Nguyen, Yanxiu Li, Jessica Henry, Wei Zhang, Xueding Wang, Yannis M. Paulus

**Affiliations:** 1 Department of Ophthalmology and Visual Sciences, University of Michigan, Ann Arbor, MI 48105, USA; vanphucn@med.umich.edu (V.P.N.); yanxiuli@med.umich.edu (Y.L.); jeshenry@umich.edu (J.H.); 2 NTT Hi-tech Institute, Nguyen Tat Thanh University, Ho Chi Minh City 70000, Vietnam; 3 Department of Biomedical Engineering, University of Michigan, Ann Arbor, MI 48105, USA; zhawei@umich.edu (W.Z.); xdwang@umich.edu (X.W.)

**Keywords:** photoacoustic microscopy, optical coherence tomography, choroidal vascular occlusion, multimodal imaging system, spectroscopic photoacoustic microscopy

## Abstract

Photoacoustic microscopy is a novel, non-ionizing, non-invasive imaging technology that evaluates tissue absorption of short-pulsed light through the sound waves emitted by the tissue and has numerous biomedical applications. In this study, a custom-built multimodal imaging system, including photoacoustic microscopy (PAM) and optical coherence tomography (OCT), has been developed to evaluate choroidal vascular occlusion (CVO). CVO was performed on three living rabbits using laser photocoagulation. Longitudinal imaging of CVO was obtained using multiple imaging tools such as color fundus photography, fluorescein angiography, indocyanine green angiography (ICGA), OCT, and PAM. PAM images were acquired at different wavelengths, ranging from 532 to 700 nm. The results demonstrate that the CVO was clearly observed on PAM in both two dimensions (2D) and 3D with high resolution longitudinally over 28 days. In addition, the location and margin of the CVO were distinguished from the surrounding choroidal vasculature after the injection of ICG contrast agent. PAM imaging was achieved using a laser energy of approximately 80 nJ, which is about half of the American National Standards Institute safety limit. The proposed imaging technique may provide a potential tool for the evaluation of different chorioretinal vascular disease pathogeneses and other biological studies.

## 1. Introduction

Choroidal vascular occlusion (CVO), or choroidal ischemia, is disruption of the blood flow to the choroid of the eye that can cause irreversible, severe vision loss [1,2,3]. CVO may result as a complication from photodynamic therapy, thermal laser photocoagulation, or ocular compression. CVO can be diagnosed by indocyanine green angiography (ICGA) and fluorescein angiography (FA) [4]. The absence of fluorescent dye in the choroidal vasculature or choriocapillaris is evidence of occlusion. However, FA and ICGA are invasive procedures requiring the use of exogenous contrast agents. Although fluorescent dyes are approved by the US Food and Drug Administration (FDA), the dye may cause side-effects such as nausea or vomiting in 5–16% of patients and can cause severe allergic reactions including anaphylaxis and death [5,6,7,8].

Optical coherence tomography (OCT) and OCT angiography (OCTA) are routinely used in retina clinics for depth-resolved evaluation of retinal anatomy and vasculature [9]. OCT and OCTA enable direct evaluation of retinal vasculature (without using exogenous contrast agents) with high resolution and contrast [10,11]. However, the depth of penetration of OCT is limited in the evaluation of the choroid [12], and OCTA cannot demonstrate leakage. Variable interscan time analysis (VISTA) has been developed to help evaluate blood flow speed [13], however a precise quantification of velocity remains elusive [14].

Photoacoustic imaging (PAI) has been explored as a novel, non-invasive, valuable imaging tool for use in several biological applications from the cellular level to organ level, with a spatial resolution of less than 10 micrometers and a penetration depth of up to 10 cm [15]. Recently, integration of PAI with other imaging techniques such as ultrasound, fluorescence, and OCT have been developed as multimodal dual or triple imaging modalities for enhanced visualization of complex biological structures [16,17]. In ophthalmology, photoacoustic microscopy (PAM) has been developed to visualize normal and abnormal retinal anatomy including retinal and choroidal microvasculature, retinal pigment epithelium (RPE), melanin, corneal neovascularization, retinal neovascularization, and choroidal neovascularization (CNV) [18,19]. PAM combines benefits of ultrasound and optical imaging, allowing for imaging of targeted tissues with high resolution and a high depth of penetration [20]. Several reports have shown that multimodal PAM and OCT, fluorescence, or scanning laser ophthalmoscopy (SLO) can improve the visualization of retinal tissues. Liu and Song et al. have integrated PAM, OCT, and SLO for the imaging of CNV, RPE, and melanin in mice [19,20,21]. In previous studies, our group has reported an integrated PAM and OCT system for label-free imaging of retinal and choroidal vessels, retinal vein occlusion, and retinal neovascularization with high resolution and high image contrast [10,22,23,24,25,26,27,28]. In addition, our current multimodal PAM and OCT system can provide high spatial resolution that allows for the identification of both normal and abnormal retinal microvasculature. These unique capabilities imply that multimodal imaging with PAM and OCT has potential in evaluating CVO.

In this study, we report the capability of a custom-built, high-resolution multimodal PAM and OCT imaging system for the visualization, characterization, and quantification of laser-induced choroidal vascular occlusion (CVO) in living rabbits over 28 days. CVO was further assessed with color fundus photography, fluorescein angiography (FA), and indocyanine green angiography (ICGA). Furthermore, spectroscopic PAM was also obtained at different wavelengths to determine the optimal excitation wavelength for imaging of CVO.

## 2. Results

**Figure 1:**
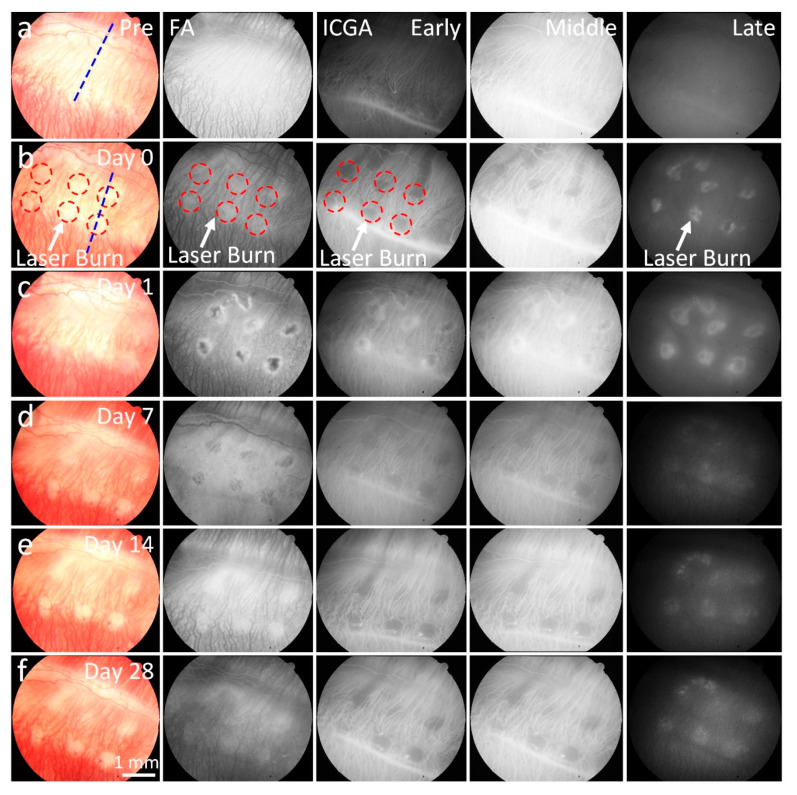
In vivo color fundus photography, fluorescein angiography (FA) and indocyanine green angiography (ICGA) imaging from the right eye of a rabbit with choroidal vascular occlusion (CVO). (**a**) Baseline color fundus, FA, and ICGA (early, middle, and late phases) images acquired before laser-induced CVO, showing clear microvasculature. (**b**–**f**) Images of CVO acquired post laser illumination at different time points: day 1, 7, 14, and 28. Red circles indicate the position of CVO; blue dotted line shows the selected scanning line for OCT. These images show reduced color intensity (color fundus) or fluorescent intensities (FA and ICGA) at the corresponding location of CVO (white arrows).

The CVO model was monitored with color fundus photography, FA, ICGA, PAM, and OCT imaging longitudinally over a period of 28 days. Figure 1a shows color fundus photography images of retinal vessels (RVs), choroidal vessels (CVs), and CVO acquired at different times. The color fundus image before laser treatment shows normal retinal structures. In contrast, there was a significant loss of choroidal vessels at the laser injury sites post laser irradiation (white dotted circles). The CVO positions were clearly visualized due to the change of color intensity on the fundus images. Note that no severe hemorrhage was observed on the fundus images post laser illumination. Figure 1b–f demonstrates FA and ICGA images acquired at different times (early, middle, and late phase). These images illustrate the morphology of the retinal microvasculature. There was no evidence of blood perfusion or reperfusion at the location of laser injury post CVO up to 28 days, indicating that choroidal vessels were disrupted in this region for at least 28 days. Laser scars were observed around the laser lesions from the leakage of fluorescent molecules on the FA and ICGA at day 14 post treatment and remained for up to 28 days (white arrows). The choroidal vessel network around the laser injury sites was not significantly changed, and no evidence of newly developed vessels was observed.

**Figure 2:**
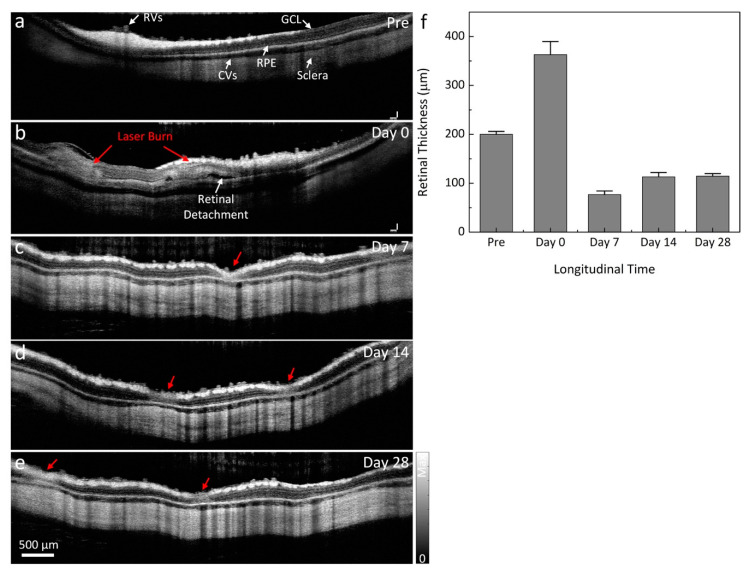
Optical coherence tomography (OCT) visualization of CVO. (**a**) B-scan OCT images acquired along the selected blue scanning line from (**a**). This image demonstrates retinal vessels (RVs), choroidal vessels (CVs), the ganglion cell layer (GCL), retinal pigment epithelium (RPE), and sclera. These layers are intact. (**b**–**e**) Two dimensional (2D) cross-sectional OCT images acquired after laser illumination. Retinal detachment appeared after CVO (white arrow). The normal retinal morphology is dramatically altered at the laser injury sites (red arrows). (**f**) Graph of the measured retinal thickness profile from the laser injury sites. This shows the dynamic changes of retinal thickness over time.

To evaluate the changes to the retinal layers post-laser, OCT images were employed. Figure 2 shows the longitudinal in vivo OCT visualization of the laser injury sites. Cross-sectional B-scan OCT images were acquired along the dotted lines shown in Figure 1a,b. The OCT image before laser treatment demonstrates different retinal layers, such as the retinal vessels, nerve fiber layer, ganglion cell layer (GCL), inner plexiform layer, inner nuclear layer, outer plexiform layer, outer nuclear layer, photoreceptors, retinal pigment epithelium (RPE), choroidal vessels, and sclera. These tissue layers were intact and were noted to be in distinct, different layers with high contrast before CVO. On the contrary, the retinal layers were severely altered post CVO. As shown in the OCT images, localized retinal detachment was observed around the laser injury sites. The retinal detachment occurred at day 0 (Figure 2b) post CVO with the accumulation of subretinal fluid (white arrow). Figure 2f shows the thickness profile of CVO measured from a group of three rabbits (*n* = 3). The retinal thickness was significantly changed post CVO. The retinal tissue had an average thickness of 362.85 ± 26.82 µm at day 0 post treatment, which was a 181.3% increase when compared to that of before CVO (thickness = 200.15 ± 5.80 µm for pre-CVO). The retinal thickness significantly reduced over time with a thickness of 76.76 ± 7.34 µm at day 7 (*n* = 3).

**Figure 3:**
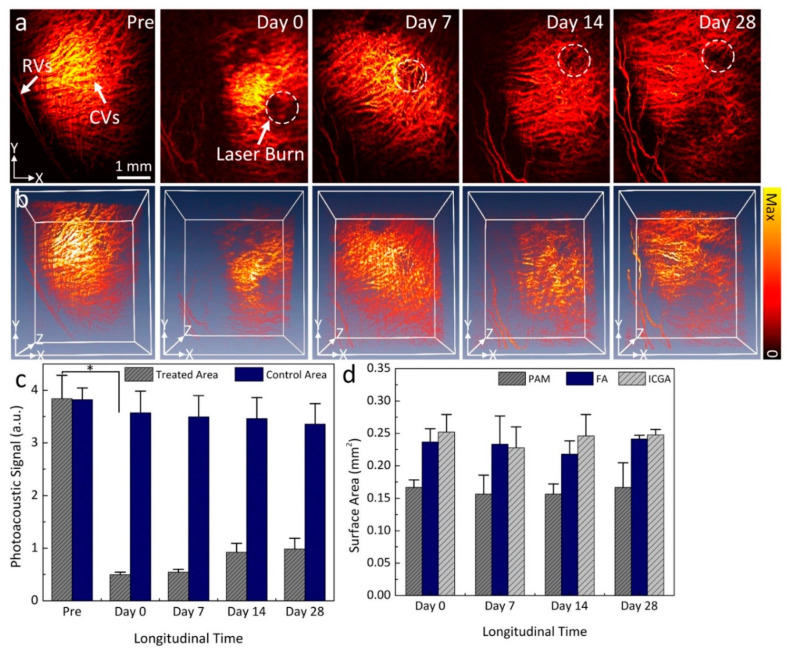
Longitudinal visualization of CVO before and after laser irradiation. (**a**) PAM images obtained at different time points over a period of 28 days post laser-induced CVO. Retinal vessels (RVs) and choroidal vessels (CVs) (white arrows) were visualized on the PAM images obtained before laser irradiation. White dotted circles demonstrate the location of CVO. At the CVO position, the image contrast was reduced in comparison to the surrounding choroidal vessels. (**b**) Three-dimensional (3D) volumetric visualization of PAM images. (**c**) Graph of PAM signal amplitude profiles. (**d**) Surface area of CVO measured by different imaging modalities: PAM, FA, and ICGA. Data presents as mean ± deviation (*n* = 3; *: *p* < 0.001).

Figure 3a shows the in vivo long-term visualization of CVO using PAM. In this experiment, the CVO was imaged at various time points at an excitation wavelength of 578 nm. The PAM image acquired before CVO was used as a control. The PAM image obtained before CVO clearly illustrates the anatomy of the retinal and choroidal vessels with great contrast. The PAM contrast was provided by the strong optical absorption of hemoglobin (Hb) within vessels (i.e., µ_Hb_ = 270.56 cm^−1^). The diameter of choroidal vessels was estimated to range from 38 µm to 130 µm, and the diameter of the retinal vessels was estimated to be 70.3 ± 5.4 µm. This data is consistent with a previous report by Tian el al. [28]. After CVO, the PAM image shows the dynamic changes to choroidal vessels over time, with the location of CVO clearly observed at each time point. To better visualize the entire CVO, three-dimensional volumetric PAM images were rendered from the acquired PAM data as depicted in Figure 3b. These images illustrate the 3D structure of CVO along with the major retinal and choroidal vessels. This implies that PAM imaging has potential for monitoring of CVO over time. By performing segmentation to extract the margin of CVO and to precisely determine the PAM signal amplitude at different regions of interest (ROI) such as CVO and choroidal vessels, the PAM signal amplitudes at CVO reduced by 7.8 times at day 0 post CVO when compared to the control vessels (Figure 3c). The decreased signal likely occured due to a reduction in hemoglobin at that position, resulting in clear visualization the margin of CVO (PA_Amplitude_ = 3.84 ± 0.44 (a.u.) for CVO vs. 0.5 ± 0.05 (a.u.) for control choroidal vessels). Note that the PA signal amplitudes decreased 7.0-, 4.2-, and 3.9-fold at day 7, 14, and 28 respectively. This illustrates that the choroidal vessels are affected significantly over time. In contrast, the PA signal measurement from control choroidal vessels areas are slightly reduced post CVO.

To further evaluate the capability of PAM for tracking CVO, the surface area of CVO was quantified on the maximum intensity projection PAM images and compared with the one measured from color fundus photography, FA, and ICGA images. Figure 3d shows the graph of surface area profiles measured from the isolated CVO areas at each time point. The results demonstrate that the surface area (SA) measured from PAM images was estimated to be 0.17 ± 0.01 mm^2^ at day 0 and did not change over a period of 28 days (i.e., SA = 0.16 ± 0.03, 0.16 ± 0.02, and 0.17 ± 0.04 mm^2^ for day 7, 14, and 28, respectively; *p* < 0.001). It was noted that this result is quite similar to that of the one measured from color fundus, FA, and ICGA images. For example, the measured SA is 0.24 ± 0.02 mm^2^ for FA and 0.25 ± 0.03 mm^2^ for ICGA.

**Figure 4:**
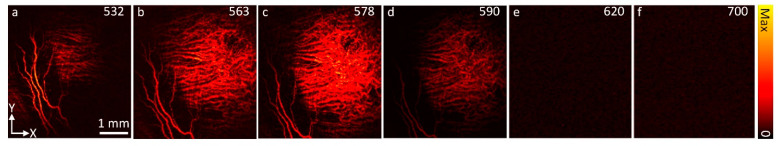
In vivo spectroscopic PAM images acquired at different excitation wavelengths, ranging from 532 nm to 700 nm. The strongest PAM contrast occurred at 563 and 578 nm. On the contrary, longer wavelengths of 620 nm and 700 nm exhibited low PAM contrast.

**Figure 5:**
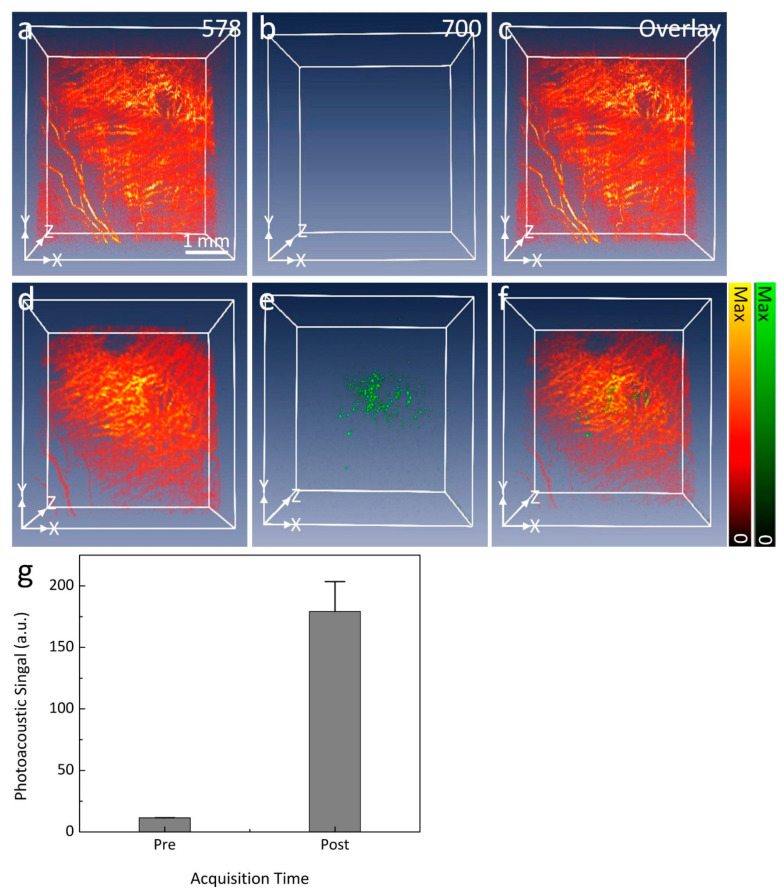
In vivo PAM visualization of CVO. (**a**–**c**) Three-dimensional PAM images of CVO acquired at two different wavelengths (578 and 700 nm) before the intravenous injection of ICG dye. No PAM signal was observed on the PAM images obtained at 700 nm. (**d**–**f**) Three-dimensional PAM images of CVO obtained 15 min after injection. The location of CVO and scars were observed and illustrate high image contrast at the wavelength of 700 nm in comparison with before injection. Overlay 3D image (**f**) demonstrates the evaluation of CVO when compared to the surrounding choroidal microvasculature. (**g**) A significant increase in photoacoustic signal at 700 nm after intravenous administration of ICG can be seen.

To selectively identify the margin of CVO, spectroscopic PAM imaging was implemented using an excitation wavelength ranging from 532 to 700 nm (Figure 4). The margin of CVO was clearly visible on PAM with great contrast acquired at 578 nm as a result of the strong absorption coefficient of hemoglobin (i.e., µ_Hb_ = 270.56 cm^−1^). In contrast, PAM images acquired at shorter or longer wavelengths show less contrast due to low optical absorption of Hb at those wavelengths (i.e., µ_Hb_ = 77.76, and 17.28 cm^−1^ at 590 and 620 nm, respectively) [29]. To distinguish laser scars around CVO, PAM images were acquired at two different wavelengths (578 and 700 nm) following the intravenous injection of indocyanine green (ICG) fluorescence dye. PAM imaging was acquired immediately after the injection of ICG. As shown in Figure 5b, the PAM image acquired at 700 nm before injection of ICG shows minimal PA signal. In contrast, the location of the laser scar was clearly visible on the PAM after intravenous administration of ICG (Figure 5d). Overlay 3D imaging (Figure 5f) demonstrates better visualization of the laser scar against the surrounding choroidal vessels (pseudo-green color). By isolating the PA signal at the laser scar, the PA signal was measured and exhibited a 15.7-fold greater signal than that of before the injection due to the strong absorption of ICG dye (PA_Amplitude_ = 11.44 ± 0.13 (a.u.) for pre-injection vs. 179.22 ± 24.29 (a.u.) for post-injection) as shown in Figure 5g.

**Figure 6:**
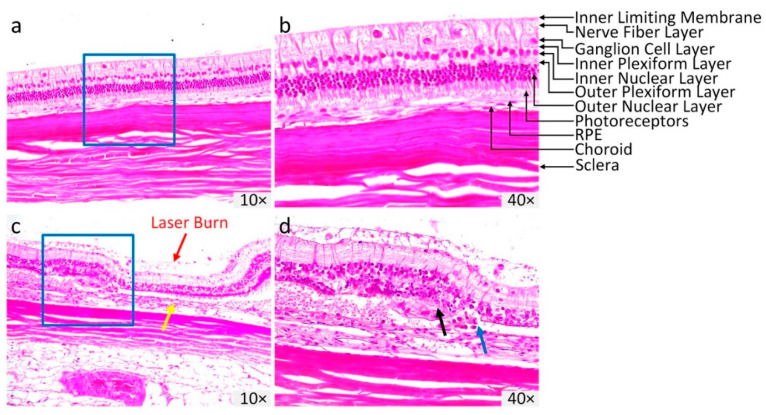
Histopathological analysis of CVO. (**a**,**b**) Hematoxylin and eosin (H&E) images acquired from control eyes without laser treatment showing the normal retinal morphology and different layers of the retina. (**c**,**d**) H&E images of CVO obtained at day 28 post laser illumination. At the laser injury site, the retinal layer demonstrated significant change in retinal morphology, with the thickness reduced from the control group. Red arrows illustrate the position of laser injury site. Magnification: 10× and 40×.

Figure 6 illustrates the hematoxylin and eosin (H&E) stained images of the control and treated retinal tissues at day 28 post CVO. The control H&E image shows normal anatomic architecture of the retina with different layers present, such as GCL, inner plexiform layer (IPL), inner nuclear layer (INL), outer plexiform layer (OPL), outer nuclear layer (ONL), photoreceptors, RPE, and choroid (Figure 6a). In contrast, the H&E image of CVO tissue exhibits a dramatic alteration in the retinal layers involving RPE loss, disorganization of the inner and outer plexiform layers, and photoreceptors (Figure 6b). The retinal thickness was thinner at about two thirds of the normal retinal thickness in CVO (i.e., thickness = 99.73 ± 0.78 µm for control vs. 70.99 ± 2.43 µm for CVO). The choroidal layer was 28% thinner at the laser injury site (yellow arrow) compared to the control areas (thickness = 53.93 ± 0.53 µm for CVO vs. 68.82 ± 0.87 µm for control areas). In addition, disruption of Bruch’s membrane (blue arrow), and macrophages and cellular debris were observed (black arrow), illustrating the formation of a choroidal scar as evident on the ICGA image (Figure 2).

## 3. Discussion

The current study demonstrates that high-resolution multimodal photoacoustic microscopy (PAM) and optical coherence tomography (OCT) imaging can precisely visualize CVO, or choroidal ischemia, sites in rabbits in vivo without the need for an exogenous contrast agent. The choroidal vasculature in this study was occluded by laser photocoagulation. Multimodal imaging, including color fundus photography, FA, ICGA, PAM, and OCT, confirmed that CVO persisted for at least up to 28 days without reperfusion. The results also demonstrate a vascular scar that appears at day 7 post photocoagulation. The choroidal vascular scars can be distinguished from the healthy choroidal vessels using spectroscopic PAM. This is the first study illustrating PAM imaging to detect choroidal vascular occlusion and scars in large animal eyes.

Choroidal occlusion is usually visualized using indocyanine green angiography (ICGA), fluorescein angiography (FA), OCT, and OCTA [4,13]. However, some disadvantages of these imaging techniques include that FA and ICGA require an exogenous dye, and optical imaging with OCT/OCTA has difficulty visualizing deep choroidal microvasculature in the choroid layer, resulting in limited capability to detect, monitor, and quantify vascular occlusion. In contrast, PAM is a novel hybrid imaging technique that combines acoustic and optical properties, resulting in high spatial resolution, high image contrast, and depth information of the choroidal vasculature. PAM has the ability to detect blood flow and oxygen saturation, which can be used to determine the vascular occlusion based on the different optical absorption of oxygenated-hemoglobin and deoxygenated-hemoglobin [19,30]. As shown in Figure 4c, the PA signal amplitudes at the occlusion position was significantly reduced when compared to the adjacent choroidal microvasculature. The change of PAM signal amplitude demonstrated the location of the occlusion due to the reduction in optical absorption of hemoglobin. This can be explained by the lack of hemoglobin within the choroidal vessels, resulting in reduced PA signal. Prah et al. have shown that oxygenated hemoglobin has an absorption coefficient that is 140% higher than that of deoxygenated-hemoglobin (µa = 270.56 cm^−1^ for oxygenated-hemoglobin vs. µa = 199.91 cm^−1^ for deoxygenated hemoglobin at 580 nm).

An advantage of multimodal imaging is that it can obtain 3D volumetric images with a field of view of 4 × 4 mm^2^ and resolution of 256 × 256 pixels within 65 s. High scanning speed plays an important role in ocular imaging because the fixation time of the eye is short, leading to motion artifacts which may cause reduce image quality. The acquisition time is limited by the repetition rate of the laser. The slow repetition rate of the optical parametric oscillator (OPO) could be solved by using a laser source with a higher repetition rate. This is the first study reporting the visualization of CVO in large animal eyes using multimodal PAM and OCT imaging. Several groups have developed CVO models in rodents such as mice and rats [9,31]. One disadvantage of these studies is that the eyeball of these animals is small in comparison to human eyeballs (~3 mm for mice, ~6 mm for rats vs. ~23 mm for human), leading to limited clinical applicability. Our group has successfully demonstrated retinal vein occlusion in rabbits using laser photocoagulation with Rose Bengal [10,22]. However, this model can have side-effects on the retina after laser irradiation such as hemorrhage, the treatment area being larger than the laser incident beam size, or the retinal vessels becoming dilated and tortuous. Thus, the proposed model may be helpful for the study of choroidal vascular occlusion pathogenesis.

## 4. Materials and Methods

### 4.1. Multimodal Imaging System

**Figure 7:**
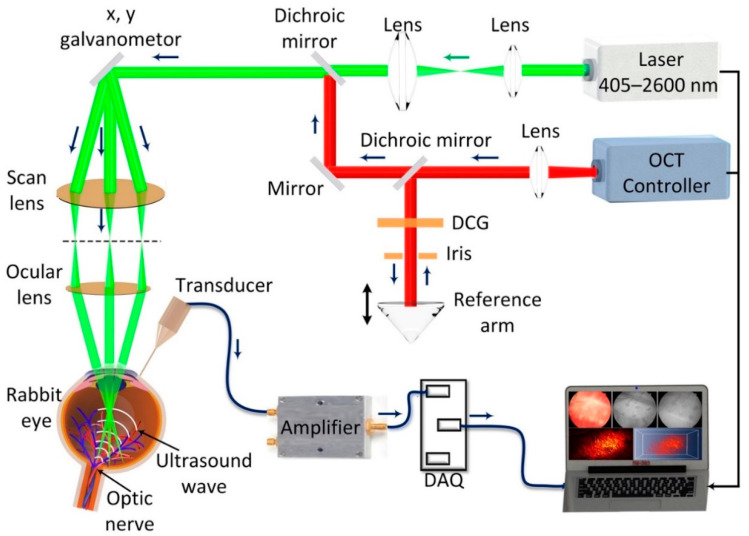
Schematic of the high resolution, multimodal photoacoustic microscopy and optical coherence tomography imaging system. DCG: dispersion compensation glass.

A multimodal ocular imaging system was developed by integrating photoacoustic microscopy (PAM) and spectral-domain optical coherence tomography (OCT) as described previously (Figure 7) [10,22,26,32,33]. To induce a photoacoustic signal (PA), a tunable nanosecond pulsed laser light was used as a light source. The laser light was generated from an OPO pumped by a diode-pumped Q-switched neodymium-doped yttrium aluminum garnet (Nd:YAG) laser (NT-242, Ekspla, Vilnius, Lithuania) with a pulse repetition rate of 1000 Hz, pulse width of 3–6 ns, and tunable wavelength ranging from 405–2600 nm. The laser light was first collimated to form a diameter of approximately 3 mm. The collimated light was delivered to a two-dimensional galvanometer before travelling to a scan lens (f = 36 mm, OCT-LK3-BB, Thorlabs, Inc., Newton, NJ, USA), and ophthalmic lens (OL, f = 10 mm, AC080-010-B-ML, Thorlabs). An output laser beam of 2 mm was delivered to the surface of the cornea and focused on the fundus by the rabbit eye optics with an estimated retinal beam diameter of approximately 20 µm. Laser energy of approximately 80 nJ was used to illuminate the target blood vessels, which is half of the ANSI safety limit (approximately 160 nJ at 578 nm). The generated PA signal from the endogenous chromophores, such as hemoglobin and melanin, were detected by a custom-made needle shaped ultrasonic transducer with a center frequency of 27 MHz (two-way −6 dB bandwidth 60%, Optosonic Inc., Arcadia, CA, USA). The detected PA signal was then amplified by a low-noise amplifier (gain 57 dB, AU-1647, L3 Narda-MITEQ, NY, USA) and digitized by a high-speed digitizer at a sampling rate of 200 MS/s (PX1500-4, Signatec Inc., Newport Beach, CA, USA). The transducer was positioned in contact with the conjunctiva. Two-dimensional (2D) and three-dimensional (3D) volumetric PAM images were rendered from the PA data. The lateral and axial resolutions are 4.1 and 37.0 µm, respectively.

Optical coherence tomography (OCT) imaging was developed from a Ganymede-II-HR OCT system (Thorlabs, Newton, NJ) by adding an ocular lens after the scan lens and dispersion compensation glass in the reference arm [27]. The OCT system has a center wavelength of 905 nm by combining two superluminescent light emitting diodes with center wavelengths of 845 nm (bandwidth = 61.9 nm) and 932 nm (bandwidth = 93.5 nm). The average power of the OCT probing light was 0.8 mW. A single mode fiber was used to deliver the light from the light source and coaxially aligned with the PAM system. The lateral and axial resolutions are 3.8 µm and 4.0 µm, respectively. The system can achieve an imaging depth of 1.9 mm.

### 4.2. Animal Model Preparation

Animal experiments were performed in accordance with the guideline of the Association for Research in Vision and Ophthalmology (ARVO) Statement on the Use of Laboratory Animals in Ophthalmic and Vision Research. The in vivo experimental protocol was approved by the Institutional Animal Care and Use Committee (IACUC) of the University of Michigan (Protocol number: PRO00008566, PI: Y. Paulus). Rabbits were anesthetized by intramuscular injection of ketamine (40 mg/kg, 100 mg/mL) (JHP Pharmaceuticals, Rochester, MI, USA) and xylazine (5 mg/kg, 100 mg/mL) (Anased^®^Boise, ID, USA). The rabbit’s pupils were dilated using tropicamide 1% ophthalmic (Akorn, Decatur, IL, USA) and phenylephrine hydrochloride 2.5% ophthalmic (Bausch & Lomb Pharmaceuticals, Tampa, FL, USA). For topical anesthesia, a drop of topical tetracaine 0.5% was applied on the cornea. To prevent corneal dehydration, a drop of eyedrop solution (Systane, Alcon Inc., TX, USA) was applied every minute during the experiment. To maintain anesthesia, a vaporized isoflurane anesthetic (1 L/min oxygen and 0.75% isoflurane) (Surgivet, MN, USA) was used along with the injection of 1/3 dose of ketamine. Animal vitals such as mucous membrane color, heart rate, respiratory rate, and rectal temperature were monitored and documented every 15 min.

### 4.3. Choroidal Vascular Occlusion (CVO) Model

The choroidal vascular occlusion (CVO) model was implemented using laser photocoagulation. Six New Zealand white rabbits (both genders) with a weight of 2.4–3.0 kg and an age of 2–4 months received laser illumination. The rabbits were generously donated from the Center for Advanced Models and Translational Sciences and Therapeutics (CAMTraST) at the University of Michigan Medical School. To induce CVO, a contact lens (Volk H-R Wide Field, laser spot 2× magnification, Volk Optical Inc., Mentor, OH, USA) was placed on the cornea of the rabbit eye. Gonak Hypromellose Ophthalmic Demulcent Solution 2.5% (Akorn, Lake Forest, IL, USA) was placed on the surface of the contact lens for coupling between the incident laser light and the cornea. Then, the rabbit eye was irradiated with a 532 nm green light laser (Vitra 532 nm, Quantel Medical, Cournon d’Auvergne, France) at a power of 450 mW to create the CVO model using a Zeiss SL 130 slit lamp (Carl Zeiss Meditec, Jena, Germany), to which the Vitra photocoagulator was connected. The laser spot size was approximately 300 µm in aerial diameter, and the pulse duration was 500 ms per spot. Twelve shots of the laser were illuminated into the eye at different positions.

### 4.4. Follow-Up of CVO Model

After laser illumination, all the treated animals were imaged with color fundus photography, FA, ICGA, PAM and OCT and followed up for 28 days with serial imaging.

### 4.5. Color Fundus Photography, FA and ICGA

The Topcon 50EX system was used to acquire the color fundus photography, FA, and ICGA images (Topcon Corporation, Tokyo, Japan). The color fundus photography images were acquired before and after the CVO model to examine the change in choroidal vessels as well as to quantify blood perfusion within the vessels. Following color fundus imaging, FA and ICGA were obtained to assess the choroidal vascular occlusion. FA and ICGA used the same Topcon 50EX system by changing the appropriate excitation and emission filters. To acquire FA images, 10% fluorescein sodium at a dose of 0.2 mL (Akorn, Lake Forest, IL, USA) was intravenously administered in the rabbit marginal ear vein. Sequential FA images were acquired immediately after injection. Late phase FA images were obtained every minute over a period of 15 min. ICGA images were acquired by the intravenous injection of ICG dye (Patheon Italia S.p.A., MB, Italy). Each rabbit was injected with 0.2 mL ICG at concentration of 2.5 mg/mL. Early, middle and late phase ICGA were achieved.

### 4.6. In Vivo PAM and OCT Imaging

All rabbits with induced CVO were imaged with PAM and OCT before and after laser illumination. To acquire PAM and OCT images, the anesthetized rabbits were placed on two different stabilization platforms to avoid motion artifacts and to adjust the position of the regions of interest (ROI) for acquiring PAM and OCT images during the in vivo experiments. After finding the ROI, B-scan OCT images were acquired. Each OCT image has a resolution of 512 × 1024 A-lines. The A-line acquisition rate was 36 kHz. After OCT imaging, the system was switched to PAM mode to acquire PAM images. The system was controlled by Matlab2019b (MathWorks, MA, USA). To acquire PAM images, the ultrasonic transducer was placed in contact with the conjunctiva of the rabbit eye. CVO was monitored over a period of 28 days after CVO to quantify the dynamic change of the choroidal vessels (i.e., occluded, non-perfusion, re-perfusion, and size of the choroidal lesions). Three-dimensional volumetric PAM images were acquired by raster scanning along x- and y-axes using an optical scanning galvanometer. It took about 65 s to achieve a volumetric image with a field of view of 4 × 4 mm^2^ and resolution of 256 × 256 pixels. The retinal thickness and laser injury depth were determined using ImageJ software (NIH, USA).

### 4.7. Histological Analysis

Histological analysis was performed to examine the CVO and retinal damage. The rabbits were euthanized at day 28 post-photocoagulation. To euthanize the rabbits, euthanasia solution (Beuthanasia-D, 0.22 mg/kg, 50 mg/mL, Vetone, ID, USA) was injected into the rabbit intravenously. The eyeballs were harvested and fixed in Davidson’s fixative solution for 24 h. The sample was then cut into 5 mm pieces and embedded in paraffin. The sample was sectioned to a thickness of 4 µm using a Leica Autostainer XL (Leica Biosystems, Nussloch, Germany) and stained with hematoxylin and eosin (H&E). The H&E slides were observed using a Leica DM600 light microscope (Leica Biosystems, Nussloch, Germany) and the images were captured using a BF450C camera.

## 5. Conclusions

In summary, the current study provides evidence that high resolution multimodal PAM and OCT imaging is a valuable imaging tool for the detection and monitoring of choroidal vascular occlusion (CVO) in large animal models. The location and margin of CVO could be distinguished from the surrounding healthy microvasculature with high contrast using the integrated PAM and OCT system. Particularly, B-scan OCT provided details of the different retinal layers which supplements vascular information from PAM imaging. This allows us to precisely visualize and differentiate the CVO depth in the choroidal layer.
